# Extracavitary Primary Effusion Lymphoma in an HIV‐Negative Patient With Aberrant Immunophenotypic Features

**DOI:** 10.1002/jha2.70443

**Published:** 2026-10-22

**Authors:** Gabriel Lopes Centoducatte, Mariana Di Giuseppe Deininger, Irene Lorand‐Metze, Guilherme Rossi Assis‐Mendonça

**Affiliations:** ^1^ Department of Pathology Anatomic Pathology Section, State University of Campinas, Rua Vital Brasil, 80, Cidade Universitária “Zeferino Vaz” Campinas São Paulo Brazil; ^2^ Laboratory of Anatomic Pathology Gastrocentro, State University of Campinas, Rua Carlos Chagas, 420, Cidade Universitária “Zeferino Vaz” Campinas São Paulo Brazil; ^3^ Hemocentro, State University of Campinas R. Albert Sabin, s/ n°. Cidade Universitária “Zeferino Vaz” Campinas São Paulo Brazil

**Keywords:** extracavitary primary effusion lymphoma, Epstein‐Barr virus, gastric lymphoma, human herpesvirus 8, primary effusion lymphoma

1

A 61‐year‐old male with a history of chronic hepatitis B virus (HBV) infection, receiving regular treatment with oseltamivir, presented with upper gastrointestinal bleeding. Laboratory investigations revealed normocytic, normochromic anaemia (haemoglobin: 7.1 g/dL). Serologic testing showed nonreactive HBsAg, reactive anti‐HBc and anti‐HBs antibodies, and nonreactive anti‐HCV, anti‐HAV IgM, and anti‐HIV‐1/2 antibodies. Upper gastrointestinal endoscopy revealed an ulcero‐infiltrative lesion in the stomach involving the entire lesser curvature and anterior wall of the gastric body extending to the incisura angularis, with extensive necrosis and multiple visible vessels without active bleeding. A biopsy was obtained.

Histopathological examination demonstrated a dense, diffuse, ulcerated atypical lymphoid infiltrate involving the gastric mucosa and permeating the gastric glands. The neoplastic cells displayed immunoblastic and plasmablastic morphology, with enlarged irregular nuclei, conspicuous nucleoli, and abundant basophilic cytoplasm (hematoxylin and eosin stains, 400× and 1000×—Figure 1A,B). Immunohistochemistry showed positivity for CD43 and CD45, with partial loss of the latter. Pan‐B‐cell markers (CD20, PAX5 and CD79a) were negative, and diffuse CD30 positivity was observed (Figure 1C). Weak and focal CD3 expression was also identified (Figure 1D), whereas other T‐cell markers (CD2, CD5, CD7, CD4 and CD8) were negative. The neoplastic cells expressed MUM1 (Figure 1E) and EMA but lacked CD138 expression. ALK, granzyme B, TIA1, CD10 and BCL6 immunostains were also negative. Epstein–Barr virus (EBV) was detected by EBER in situ hybridization (Figure 1F), and human herpesvirus 8 (HHV8) was demonstrated by LANA‐1 immunohistochemistry (Figure 1G). The Ki‐67 proliferation index exceeded 95%. Based on the morphologic, immunophenotypic, and viral findings, a diagnosis of extracavitary primary effusion lymphoma (PEL) was established [1, 2].

**FIGURE 1:**
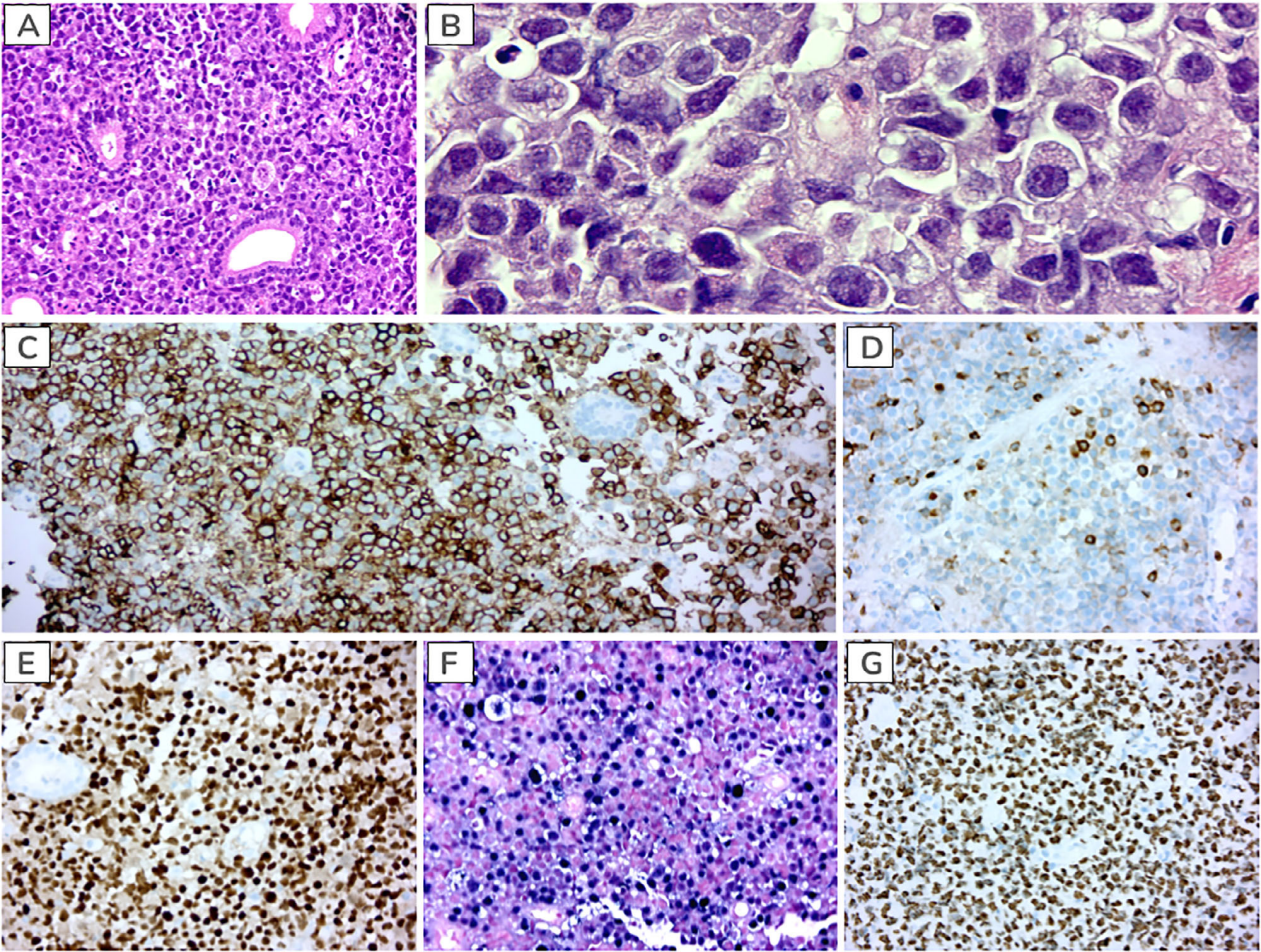
Atypical lymphoid infiltrate involving the gastric mucosa. H&E (hematoxylin and eosin) staining at 400× magnification (A) and 1000× magnification (B) demonstrates plasmablastic morphology. Immunohistochemical staining evidenced strong CD30 expression (C), weak and focal CD3 expression (D), and MUM1 positivity (E). Epstein–Barr virus (EBV) was detected by EBER in situ hybridization (F) and human herpesvirus 8 (HHV8) was detected by LANA‐1 immunohistochemistry (G).

PEL is an exceptionally rare subtype of non‐Hodgkin lymphoma in HIV‐negative patients [1, 2, 3]. It may occur in other settings of immune dysfunction, including aging and chronic infections, particularly when presenting as solid masses (extracavitary PEL) [4]. Diagnosis may be challenging because of partial loss of CD45 expression, absence of pan‐B‐cell markers, and occasional aberrant CD3 expression [1, 2]. Recognition of immunoblastic/plasmablastic morphology, together with demonstration of HHV8 and terminal B‐cell differentiation markers, is essential for diagnosis [5]. Loss of CD138, as observed in our case, represents an additional pitfall and highlights the importance of a broad immunohistochemical panel when evaluating challenging haematolymphoid neoplasms [6].

## Author Contributions

Gabriel L. Centoducatte was responsible for data collection and writing of the manuscript. Mariana D.G. Deininger was responsible for data collection, diagnostic insights and revision of the manuscript. Irene Lorand‐Metze was responsible for the patient's medical assistance and revision of the manuscript. Guilherme R.A. Mendonça was responsible for orientation, diagnostic insights and revision of the manuscript.

## Funding

The authors have nothing to report.

## Conflicts of Interest

The authors have nothing to report.

## Data Availability

Data are available upon request.
